# Worldwide Diversity of *Klebsiella pneumoniae* That Produce β-Lactamase *bla*_KPC-2_ Gene^1^

**DOI:** 10.3201/eid1609.091389

**Published:** 2010-09

**Authors:** Gaëlle Cuzon, Thierry Naas, HaVy Truong, Maria-Virginia Villegas, Karin T. Wisell, Yehuda Carmeli, Ana. C. Gales, Shiri Navon-Venezia, John P. Quinn, Patrice Nordmann

**Affiliations:** Author affiliations: Institut National de la Santé et de la Recherche Médicale, Paris, France (G. Cuzon_,_ T. Naas, H. Truong, P. Nordmann);; International Center for Medical Research and Training, Cali, Colombia (M.V. Villegas);; Swedish Institute for Infectious Disease Control, Stockholm, Sweden (K.T. Wisell);; Sourasky Medical Center, Tel Aviv, Israel (Y. Carmeli, S. Navon-Venezia);; Universidade Federal de São Paulo, São Paulo, Brazil (A.C. Gales);; John H. Stroger Jr. Hospital of Cook County, Chicago, Illinois, USA (J.P. Quinn);; Chicago Infectious Disease Research Institute, Chicago (J.P. Quinn)

**Keywords:** Klebsiella pneumoniae, β-lactamase, carbapenemase, class A, KPC, bacteria, research

## Abstract

*Klebsiella pneumoniae* isolates that produce carbapenemases (KPCs) are rapidly disseminating worldwide. To determine their genetic background, we investigated 16 *bla*_KPC-2_-harboring *K. pneumoniae* isolates from 5 countries. The isolates were multidrug resistant, possessed the *bla*_KPC-2_ gene, and differed by additional β-lactamase content. They harbored a naturally chromosome-encoded *bla* gene (*bla*_SHV-1_ [12.5%], *bla*_SHV-11_ [68.7%], or *bla*_OKP-A/B_ [18.8%]) and several acquired and plasmid-encoded genes (*bla*_TEM-1_ [81.3%], *bla*_CTX-M-2_ [31.3%], *bla*_CTX-M-12_ [12.5%], *bla*_CTX-M-15_ [18.7%], and *bla*_OXA-9_ [37.5%]). The *bla*_KPC-2_ gene was always associated with 1 of the Tn*4401* isoforms (a, b, or c). Tn*4401* was inserted on different-sized plasmids that belonged to different incompatibility groups. Several *bla*_KPC_-containing *K. pneumoniae* clones were found: 9 different pulsotypes with 1 major (sequence type 258) and 7 minor distinct allelic profiles. Different clones harboring different plasmids but having identical genetic structure, Tn*4401*, could be at the origin of the worldwide spread of this emerging resistance gene.

## Abstract 2

TOC summary: Clones harboring different plasmids with identical genetic structure could be the origin of worldwide spread.

Resistance of *Klebsiella pneumoniae* to carbapenems is mainly associated with acquired carbapenem-hydrolyzing β-lactamases (*1*). These β-lactamases can be metallo β-lactamases (IMP, VIM), expanded-spectrum oxacillinases (OXA-48), or Ambler class A enzymes (NMCA, IMI, SME, GES, and KPC) (*1**–**4*). The most common class A carbapenemases in *K. pneumoniae* are the *K. pneumoniae* carbapenemases (KPCs) (*4*). KPCs in carbapenem-resistant *K. pneumoniae* strains were first reported in 2001 in North Carolina (*5*), and until 2005, the geographic distribution of these enzymes in *Enterobacteriaceae*, including *K. pneumoniae,* was limited to the eastern part of the United States (*5**,**6*). Now, KPC-producing *K. pneumoniae* isolates are frequently identified among nosocomial pathogens (*7*). Recently, dissemination of a single major clone of KPC-producing *K. pneumoniae* (sequence type [ST] 258) in the eastern United States has been suggested (*8*). KPCs have been observed more rarely among other gram-negative rods such as *Pseudomonas* spp (*9**,**10*).

Outside the United States, KPC-producing *K. pneumoniae* are also being reported more often. The first case of KPC-producing *K. pneumoniae* infection was reported in 2005 in France and had a US origin (*11*). The first outbreak of KPC-producing *K. pneumoniae* outside the United States was in Israel (*12*). In South America, dissemination of KPC-producing *K. pneumoniae* was initially reported in 2006 in Colombia (*13*) and then in Brazil and Argentina (*14**,**15*). KPC enzymes have also been identified in *P. aeruginosa* isolates from Colombia (*16*). In the People’s Republic of China, KPC enzymes in several enterobacterial species are being increasingly reported (*17*). Finally, in Europe a few cases of KPC-producing *K. pneumoniae* infection have been described, but in Greece, outbreaks have occurred (*18*). In Europe, different variants of KPCs (KPC-2 and KPC-3) have been described; some patients carrying KPC-positive isolates had been transferred from the United States, Israel, or Greece (*19**–**21*).

Reports of this β-lactamase being found in novel locations are increasing worldwide, probably signaling active spread. The genetic element carrying the *bla*_KPC-2_ gene, Tn*4401*, was recently elucidated (*22*). Three isoforms of this Tn*3*-like transposon (a, b, and c) are known. Several other genetic environments of *bla*_KPC_ gene have been described; other insertion sequences have been found upstream of the *bla*_KPC_ gene (*23**,**24*). Nevertheless, the downstream sequences of the *bla*_KPC_ gene matched perfectly with Tn*4401*, which suggests that these insertion sequences have been inserted into Tn*4401*.

Insertion sequences may play major roles in the evolution of Tn*4401*, but little information is available about the bacterial strains and the plasmids that may explain this rapid spread. Our goal, therefore, was to characterize the genetic background of several *bla*_KPC-2_- harboring *K. pneumoniae* isolates from various geographic origins.

## Materials and Methods

### Bacterial Strains

*K. pneumoniae* isolates used in this study and their origin are listed in Table 1 (*11**,**13**,**16**,**21**,**25*). Electrocompetent *Escherichia coli* DH10B (Invitrogen, Eragny, France) was used as a recipient in electroporation experiments. *E. coli* J53Az^R^, which is resistant to sodium azide, was used for conjugation experiments. *E. coli* 50192 was used as a reference strain for plasmid extraction (*22*).

**Table 1:** Geographic origin and structure of Tn*4401* and other β-lactamases of *Klebsiella pneumoniae* isolates*

Isolate no.	Isolate type	Origin	PCR result									
			KPC-2	Tn*4401*					Other β-lactamases			
				TnpA	ISKPN7	ISKPN6	Deletion, bp		SHV	TEM	CTX-M	OXA
1	YC	USA	+	+	+	+	–100		SHV-11	TEM-1	–	OXA-9
2	GR	Greece	+	+	+	+	–100		SHV-11	TEM-1	–	OXA-9
3	K271	Sweden	+	+	+	+	–100		SHV-11	TEM-1	–	OXA-9
4	KN2303	Colombia	+	+	+	+	None		SHV-11	–	–	–
5	KN633	Colombia	+	+	+	+	None		OKP-A	TEM-1	CTX-M-12	–
6	INC H1521-6	Colombia	+	+	+	+	None		SHV-1	TEM-1	CTX-M-15	–
7	INC H1516-6	Colombia	+	+	+	+	None		SHV-1	TEM-1	CTX-M-15	–
8	HPTU 27635	Colombia	+	+	+	+	None		OKP-B	–	–	–
9	HPTU 2020532	Colombia	+	+	+	+	None		OKP-A	TEM-1	CTX-M-12	–
10	A28006	Brazil	+	+	+	+	None		SHV-11	TEM-1	CTX-M-2	–
11	A28008	Brazil	+	+	+	+	None		SHV-11	TEM-1	CTX-M-2	–
12	A28009	Brazil	+	+	+	+	None		SHV-11	TEM-1	CTX-M-2	–
13	A28011	Brazil	+	+	+	+	None		SHV-11	TEM-1	CTX-M-2	OXA-9
14	A33504	Brazil	+	+	+	+	None		SHV-11	TEM-1	CTX-M-2	OXA-9
15	475	Israel	+	+	+	+	–200		SHV-11	–	CTX-M-15	–
16	588	Israel	+	+	+	+	–200		SHV-11	TEM-1	–	OXA-9

*KPC, *K. pneumoniae* carbapenemase.

### Antibiograms and MIC Determinations

Antibiograms were created by using the disk-diffusion method on Mueller-Hinton agar (Bio-Rad Laboratories, Marnes-La-Coquette, France), and susceptibility break points were determined as previously described and interpreted as recommended by the Clinical and Laboratory Standards Institute (*22**,**26*). All plates were incubated at 37°C for 18 h. MICs of β-lactams were determined by using the Etest technique (bioMérieux, Marcy l’Etoile, France).

### Electroporation and Plasmid Extraction

Direct transfer of resistance into azide-resistant *E. coli* J53 was attempted as reported (*22*). Plasmids were introduced by electroporation into *E. coli* DH10B (*22*) by using a Gene Pulser II (Bio-Rad Laboratories).

Plasmid DNA was extracted by using a QIAGEN Plasmid Maxi Kit (QIAGEN, Courtaboeuf, France) and analyzed by agarose gel electrophoresis (Invitrogen, Paris, France). Natural plasmids were extracted by using the Kieser extraction method (*27*) and subsequently analyzed by electrophoresis on a 0.7% agarose gel.

### Hybridization

DNA–DNA hybridization was performed as described by Sambrook et al. (*28*) with Southern transfer of an agarose gel containing Kieser method–extracted total DNA. The probe consisted of a 796-bp PCR-generated fragment from recombinant plasmid pRYC-1 (*22*) and was internal to the *bla*_KPC-2_ gene. Labeling of the probe and detection of signal were conducted by using an ECL nonradioactive labeling and detection kit according to the manufacturer's instructions (Amersham Biosciences, Orsay, France).

### PCR Amplification and Sequencing

The *bla*_CTX-M_-, *bla*_SHV_-, *bla*_TEM_-, and *bla*_OXA-1/9_-like genes were searched for and characterized as described (*21*). PCR experiments were performed on an ABI 2700 thermocycler (Applied Biosystems, Les Ulis, France) by using laboratory-designed primers (Table 2). PCR products were then analyzed on agarose gel and sequenced.

**Table 2:** Primers used for PCR of *Klebsiella pneumoniae* isolates producing β-lactamase *bla*_KPC-2_ gene*

Primer name	Primer no.†	Sequence, 5′ → 3′
KpcA	1	CTGTCTTGTCTCTCATGGCC
KpcB	2	CCTCGCTGTGCTTGTCATCC
4281	3	GGCACGGCAAATGACTA
4714	4	GAAGATGCCAAGGTCAATGC
EcoRIout	5	CACCCGACCTGGACGAACTA
3′YCEnd	6	GCATCAAACGGAAGCAAAAG
3781L	7	CACAGCGGCAGCAAGAAAGC
3098U	8	TGACCCTGAGCGGCGAAAGC
905L	9	GCGACCGGTCAGTTCCTTCT
816U	10	CACCTACACCACGACGAACC
141R-6	11	TCACCGGCCCTCACCTTTGG
5′endYC	12	CTTAGCAAATGTGGTGAACG
Pre-SHV-5 U	–	GGTCAGCGCGAGAAGCATCC
Pre-SHV-5 L	–	AAATAGCGTTCATCGTCAAT
Pre-TEM 1	–	GTATCCGCTCATGAGACAATA
Pre-TEM 2	–	TCTAAAGTATATATGAGTAAACTTGGTCTG
OXA-9 A	–	TTCGTTTCCGCCACTCTCCC
OXA-9 B	–	ACGAGAATATCCTCTCGTGC
CTX-M A	–	CGCTTTGCGATGTGTCAG
CTX-M B	–	ACC GCG ATA TCG TTG GT

**Figure 1:**
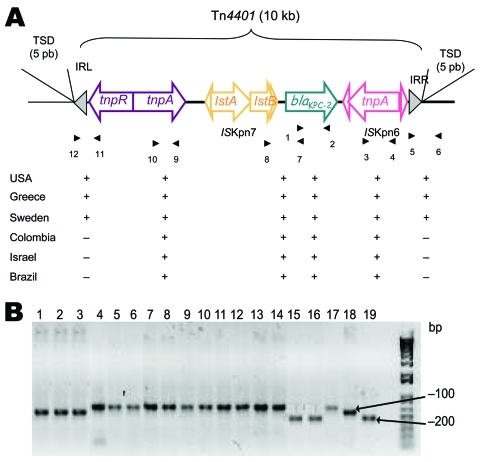
A) Schematic representation of Tn*4401* isoforms on plamids of *Klebsiella pneumoniae* isolates that produce *K. pneumoniae* carbapenemases (KPCs). Genes and their corresponding transcription orientations are indicated by horizontal arrows. Gray triangles represent the inverted repeats left (IRL) and right (IRR) of Tn*4401*. Small and empty triangles represent the inverted repeats of IS*Kpn6* and IS*Kpn7*. Target site duplications (TSD) are indicated above the sequence. Primers listed in Table 2 are shown below, with results of PCRs for each isolate. B) PCR results with primers 7 and 8 (Table 2). Lane 1, *K. pneumoniae* YC (*11*); lane 2, *K. pneumoniae* GR (*21*); lane 3, *K. pneumoniae* K271 (*25*); lane 4, *K. pneumoniae* KN2303 (*13*); lane 5, *K. pneumoniae* KN633 (*13*); lane 6, *K. pneumoniae* INC H1521-6; lane 7, *K. pneumoniae* INC H1516-6; lane 8, *K. pneumoniae* HPTU 27635; lane 9, *K. pneumoniae* HPTU 2020532; lane 10, *K. pneumoniae* A28006 (*16*); lane 11, *K. pneumoniae* A28008 (*16*); lane 12, *K. pneumoniae* A28009 (*16*); lane 13, *K. pneumoniae* A28011 (*16*); lane 14, *K. pneumoniae* A33504 (*16*); lane 15, *K. pneumoniae* 475; lane 16, *K. pneumoniae* 588.

*Primers from (*21*). †Numbers correspond to those in Figure 1, panel A. – indicates primer not shown in Figure 1, panel A.

Both strands of the PCR products were sequenced by using laboratory-designed primers with an automated sequencer (ABI PRISM 3100; Applied Biosystems). The nucleotide and the deduced protein sequences were analyzed by using software from the National Center for Biotechnology Information (www.ncbi.nlm.nih.gov).

### Isoelectrofocusing

Crude β-lactamase extracts, obtained as described (*21*) from 10-mL cultures of clinical isolates and their *E. coli* transconjugants or electroporants were subjected to analytical isoelectrofocusing on an ampholine-containing polyacrylamide gel, pH 3.5–9.5 (Ampholine PAG plate; GE Healthcare, Orsay, France) for 90 min at 1,500 volts, 50 milliamps, and 30 watts. The focused β-lactamases were detected by overlaying the gel with 1 mmol nitrocefin (Oxoid, Dardilly, France). Isoelectric points were determined and compared with those of known β-lactamases (*22*).

### Pulsed-field Gel Electrophoresis

Pulsed-field gel electrophoresis (PFGE) was performed by using *Xba*I (GE Healthcare) as described (*29*). *Xba*I-macrorestriction patterns were interpreted according to the recommendations of Tenover et al. (*30*).

### Multilocus Sequence Typing

Multilocus sequence typing (MLST) with 7 housekeeping genes (*rpoB*, *gapA*, *mdh*, *pgi*, *phoE*, *infB*, *and tonB*) was performed according to Diancourt et al. (*31*). Allele sequences and STs were verified at http://pubmlst.org/kpneumoniae. A different allele number was given to each distinct sequence within a locus, and a distinct ST number was attributed to each distinct combination of alleles.

### Replicon and Transposon Typing

PCR-based replicon typing of the main plasmid incompatibility groups reported for *Enterobacteriaceae* was performed as described (*32*). Genetic structures surrounding the *bla*_KPC-2_ gene were determined according to the Tn*4401* PCR-mapping scheme as described (*22*).

## Results

### Pulsotypes

Molecular typing by PFGE identified 9 major pulsotypes among the isolates (Table 3). The first pulsotype (pulsotype A) corresponded to the strains from the United States and Greece. We found 4 different pulsotypes (B–E) among strains from Colombia, which suggested polyclonal diffusion inside this country. We also identified 2 different clones among strains from Brazil (pulsotypes F and G) and from Israel (pulsotypes H and I). These results indicate much heterogeneity among KPC-producing isolates from various geographic regions.

**Table 3:** Plasmid analysis, pulsotype, and sequence type of *Klebsiella pneumoniae* isolates from 5 countries*

Isolate no.	Isolate name	Plasmids		PFGE type	MLST							ST
		Size, kb	Inc		gap	infB	mdh	pgi	phoE	rpo	tonB	
1	YC	80	FiiAS	A	3	3	1	1	1	1	79	258
2	GR	80	FiiAS	A	3	3	1	1	1	1	79	258
3	K271	80	FiiAS	A	3	3	1	1	1	1	79	258
4	KN2303	75, 35	N	B	2	1	11	1	1	1	13	337
5	KN633	12	ND	C	17	19	22†	39	34†	21	52	338
6	INC H1521-6	75	L/M	D	1	6	1	1	1	1	1	14
7	INC H1516-6	75	L/M	D	1	6	1	1	1	1	1	14
8	HPTU 27635	35	L/M	E	18	15	25†	24	11†	13	51	339
9	HPTU 2020532	12	ND	C	17	19	22†	39	34†	21	52	338
10	A28006	12	L/M	F	3	3	1	1	1	1	4	11
11	A28008	12	L/M	F	3	3	1	1	1	1	4	11
12	A28009	12	L/M	F	3	3	1	1	1	1	4	11
13	A28011	12	L/M	F	3	3	1	1	1	1	4	11
14	A33504	50	ND	G	3	3	1	1	1	1	4	11
15	475	80	N	H	3	1	1	1	1	1	43	277
16	588	70	N	I	3	3	1	1	1	1	18	340

**Figure 2:**
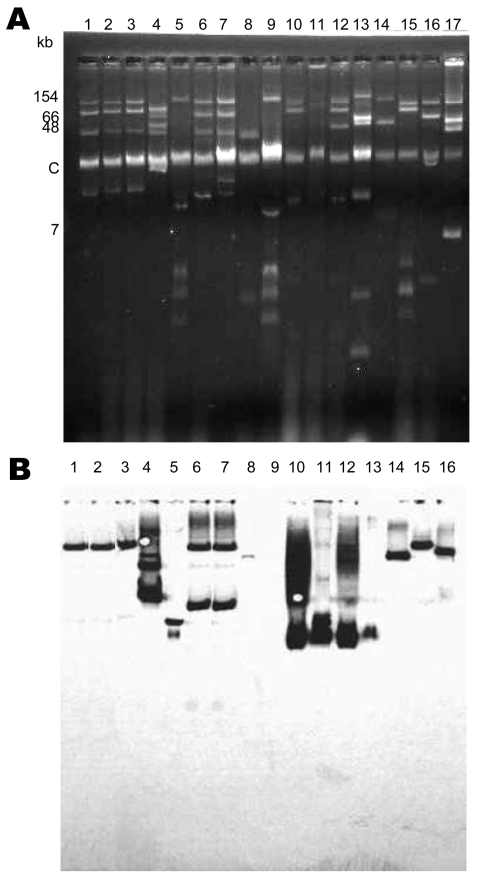
A) Plasmid extractions of culture of clinical *Klebsiella pneumoniae* isolates that produce β-lactamase *bla*_KPC-2_ gene. B) Southern hybridization of transferred plasmid extraction, conducted with an internal probe for *bla*_KPC-2_. Lane 1, *K. pneumoniae* YC (*11*); lane 2, *K. pneumoniae* GR (*21*); lane 3, *K. pneumoniae* K271 (*25*); lane 4, *K. pneumoniae* KN2303 (*13*); lane 5, *K. pneumoniae* KN633 (*13*); lane 6, *K. pneumoniae* INC H1521-6; lane 7, *K. pneumoniae* INC H1516-6; lane 8, *K. pneumoniae* HPTU 27635; lane 9, *K. pneumoniae* HPTU 2020532; lane 10, *K. pneumoniae* A28006 (*16*); lane 11, *K. pneumoniae* A28008 (*16*); lane 12, *K. pneumoniae* A28009 (*16*); lane 13, *K. pneumoniae* A28011 (*16*); lane 14, *K. pneumoniae* A33504 (*16*); lane 15, *K. pneumoniae* 475; lane 16, *K. pneumoniae* 588; and lane 17, *Escherichia coli* 50192 harboring 4 plasmids (7, 48, 66, and 154 kb).

*Countries shown in Table 1. PFGE, pulsed-field gel electrophoresis; MLST, multilocus sequence type; ST, sequence type; ND, could not be determined with the Inc primers tested. †Alleles are variants of previously described alleles (Figure 2).

MLST of the 16 isolates resulted in 8 distinct allelic profiles: ST 258 (allelic profile 3–3-1–1-1–1-79) corresponding to isolates *K. pneumoniae* YC (United States), *K. pneumoniae* GR (Greece), and *K. pneumoniae* K271 (Greece); ST 14 (allelic profile 1–6-1–1-1–1-1) corresponding to isolates *K. pneumoniae* INC H1521–6 and *K. pneumoniae* INC H1516–6 (Colombia); ST 11 (allelic profile 3–3-1–1-1–1-4) corresponding to isolates from Brazil; ST 277 (allelic profile 3–1-1–1-1–1-43) corresponding to isolate *K. pneumoniae* 475 (Israel); novel ST 337 (allelic profile 2–1-11–1-1–1-13) corresponding to isolate *K. pneumoniae* KN2303 (Colombia); ST 338 (allelic profile 17–19–22–39–34–21–52) corresponding to isolates *K. pneumoniae* KN633 and *K. pneumoniae* HPTU 2020532 (Colombia); ST 339 (allelic profile 18–15–25–24–11–13–51) corresponding to isolate *K. pneumoniae* HPTU 27635 (Colombia); and ST 340 (allelic profile 3–3-1–1-1–1-18) corresponding to isolate *K. pneumoniae* 588 (Israel). The analysis of STs by eBURST (http://pubmlst.org) showed that ST 11 and ST 340 are single-locus variants of ST 258 and that ST 277 is a double-locus variant of ST 258. These results matched perfectly with PFGE results. One isolate from Brazil (*K. pneumoniae* A33504) showed a different pattern by PFGE but the same ST (ST 11) as other isolates from the same origin, which suggests a strong genetic relatedness.

### Antimicrobial Drug Susceptibility

All isolates were resistant to penicillins and cephalosporins but showed varying levels of susceptibility to carbapenems (Table 4). Resistance to other drug classes varied among the isolates. For aminoglycosides, 2 clones (A and I) were susceptible to gentamicin only, 1 clone (H) was susceptible to amikacin only, and 3 clones (C, D, and G) were resistant to all tested aminoglycosides. Six clones (A, C, D, F, G, and I) showed resistance to fluoroquinolones. Percentages of nonsusceptible isolates to the non–β-lactam drugs were as follows: gentamicin, 75%; amikacin, 81.3%; ciprofloxacin, 81.5%; trimethoprim/sulfamethoxazole, 81.5%; and tetracycline, 87.5%. Two isolates were also resistant to colistin (*K. pneumoniae* GR and *K. pneumoniae* K271); each was from Greece, where this drug is often used (*33*).

**Table 4:** MICs of carbapenems for clinical *Klebsiella pneumoniae* isolates expressing KPC-2 β-lactamase*

Isolate type	Carbapenem MIC, mg/L		
	Imipenem	Meropenem	Ertapenem
YC	4	2	24
GR	12	6	12
K271	4	4	16
KN2303	>32	>32	>32
KN633	>32	4	>32
INC H1521-6	6	3	8
INC H1516-6	4	4	32
HPTU 27635	4	2	12
HPTU 2020532	16	16	24
A28006	16	32	24
A28008	24	16	32
A28009	>32	>32	>32
A28011	>32	>32	>32
A33504	>32	>32	>32
475	16	>32	>32
588	24	16	32

*KPC, *K. pneumoniae* carbapenemase.

### β-Lactamase Genes

Positive results of CTX-M–, TEM-, SHV-, and OXA-specific PCRs are indicated in Table 1. All isolates possessed the *bla*_KPC-2_ gene and a naturally chromosome-encoded *bla* gene: *bla*_SHV-1_ (12.5%), *bla*_SHV-11_ (68.7%), or *bla*_OKP-A/B_ (18.8%). The *bla*_OKP_ genes are 1 of the 3 families of the chromosomal β-lactamase genes found in *K. pneumoniae* (*34*) with *bla*_SHV_ and *bla*_LEN_ and share 88% similarity with *bla*_SHV-1_. *K. pneumoniae* isolates also harbored several acquired and plasmid-encoded genes: *bla*_TEM-1_ (81.3%), *bla*_CTX-M-2_ (31.3%), *bla*_CTX-M-12_ (12.5%), *bla*_CTX-M-15_ (18.7%), and *bla*_OXA-9_ (37.5%).

### Characterization Results for Tn*4401*

Primer couples specific for the different genes found on Tn*4401* (Table 2; Figure 1, panel A) obtained similar-sized fragments for all strains, which suggests that the strains have a similar genetic organization. For only 1 primer pair, hybridizing in IS*Kpn7* and *bla*_KPC_ gene (primers 7 and 8 in Figure 1, panel A), located upstream of the *bla*_KPC_ gene, an ≈100-bp (*K. pneumoniae* YC, *K. pneumoniae* GR, and *K. pneumoniae* 271) or 200-bp (*K. pneumoniae* 475 and *K. pneumoniae* 588) shorter fragment was observed, compared with the Tn*4401*b structure, thus indicating that the 3 isoforms of Tn*4401* were present in this collection of isolates (Figure 1, panel B).

To investigate the flanking sequences of Tn*4401*, we used PCR primers located in the Tn*4401* structure and in the flanking sequences derived from *K. pneumoniae* YC (*22*). PCR products of expected size were obtained for *K. pneumoniae* GR and *K. pneumoniae* K271 isolates only. For all other strains, no PCR product could be obtained, suggesting that the Tn*4401* insertion site might differ from that found in *K. pneumoniae* YC.

### Genetic Support for *bla*_KPC_ in the Isolates

The carbapenem-resistant *K. pneumoniae* isolates contained several plasmids of different sizes, ranging from <5 kb to >170 kb (Figure 2, left panel). At least 1 plasmid hybridized with an internal probe for *bla*_KPC-2_ gene in each isolate, ranging from 13 kb to 80 kb (Figure 2, right panel; Table 3). We observed 2 hybridization signals (35 kb and 75 kb) for *K. pneumoniae* KN2303, as described (*22*). Plasmid location of the *bla*_KPC_ genes was confirmed by electroporation of these plasmids into *E. coli* DH10B, but no transformant could be obtained for *K. pneumoniae* 2020532. The *E. coli* transformants had a β-lactam resistance pattern that corresponded to the expression of a *bla*_KPC_-like gene. Electroporation of 4 plasmids harboring the *bla*_KPC_-like gene into *E. coli* DH10B conferred resistance to at least an aminoglycoside molecule; pINC-H1521–6, pA33504, and p588 conferred resistance to all aminoglycosides except gentamicin, and electroporation of p475 into *E. coli* DH10B led to resistance to all aminoglycosides tested. No other antimicrobial drug resistance marker was cotransferred; the transformants remained susceptible to nalidixic acid, levofloxacin, ciprofloxacin, rifampin, tetracycline, trimethoprim/sulfamethoxazole, and colistin.

Mating-out assays showed that the ≈75–80-kb plasmids harboring *bla*_KPC-2_ from *K. pneumoniae* YC, GR, K271, and KN2303 were self-transferable to *E. coli*. The smaller plasmid from *K. pneumoniae* KN633 was not transferred to *E. coli*.

### Origin of Replication

PCR-based replicon typing of the major plasmid incompatibility groups showed that the *bla*_KPC-2_-positive plasmids belonged to at least 3 incompatibility groups (IncFIIAS, IncN, and IncL/M) (Table 3). The plasmids of *K. pneumoniae* KN633, HPTU-2020532 from Colombia and *K. pneumoniae* A33504 from Brazil gave negative results with the Inc primers tested and could not be classified into a major plasmid incompatibility group.

## Discussion

Rapid spread of KPC-producing *K. pneumoniae* is a major clinical and public health concern. These broad-spectrum β-lactamases are increasing in new locations worldwide, indicating an ongoing process. Recently, a novel Tn*3*-based transposon, Tn*4401*, was identified in nonclonally related KPC-producing *K. pneumoniae* and *P. aeruginosa* isolates (*22*). This transposon is in most recently described isolates (*20**,**35**,**36*), although a recently characterized novel variant from China had another insertion sequence inserted upstream of *bla*_KPC_ gene (*24*). Identification of Tn*4401* inserted at different loci, on different plasmids, and flanked by different 5-bp target site duplications indicates a frequent and dynamic process of transposition. It has been suggested that this novel transposon is at the origin of *bla*_KPC_-like gene acquisition and dissemination (*22*). Sixteen *K. pneumoniae* isolates that express the *bla*_KPC_ gene from 5 countries were characterized here.

PFGE and MLST showed that several clones are currently spreading in different geographic locations. In Colombia, 3 pulsotypes could be identified. Overall, among the 16 isolates, 1 major ST (258) and its derivative ST 11 seemed to predominate. In a recent study that gathered isolates from 10 US states, ST 258 accounted for 70% of isolates, according to a database of KPC-producing *K. pneumoniae* PFGE results maintained by the Centers for Disease Control and Prevention (*8*). This ST has also been identified for KPC-producing *K. pneumoniae* in Sweden (in isolates imported from Greece and Israel) and more recently in Poland (*36**,**37*). These findings suggest possible international dissemination of KPC-producing ST 258. Apparently, the *K. pneumoniae* clone that contains the extended-spectrum β-lactamase (ESBL) determinant CTX-M-15 belongs to ST 11 (*38*).

KPC-producing *K. pneumoniae* contained diverse β-lactamases. All except 2 isolates harbored at least another β-lactamase; *bla*_TEM-1_ and a *bla*_CTX-M_-type ESBLs were expressed by >80% and 62.5% of isolates, respectively. KPC producers have already been associated with other β-lactamase genes, such as the widespread ESBL gene *bla*_CTX-M-15_ (*17*). SHV ESBLs have been found among isolates, as has been described for strains from the United States (*39*) and Norway (*36*). These additional β-lactamases are likely to complicate phenotype-based identification of KPC producers. Three isolates harbored the chromosome-encoded *bla*_OKP-A/B_ genes and belonged to phylogenetic group KpII, which accounts for <10% of *K. pneumoniae* strains (*34*). Coexpression of OKP enzymes and ESBLs has rarely been reported.

Isolates also demonstrated diversity in their molecular features. In this study, the KPC-2 genes were encoded on a broad variety of plasmids, as shown by previous studies (*22**,**35*). These plasmids differed in size and incompatibility groups. Similar plasmids were observed among isolates with the same ST, whereas different plasmids were also associated with similar STs. Therefore, epidemiologic investigation of KPC producers should be performed at different molecular levels.

Tn*4401* was present in all tested strains. The overall structure of Tn*4401* seemed to be conserved, except for the 100-bp to 200-bp deletion. Of the 16 isolates, 11 encoded the full-length Tn*4401*b isoform, 3 encoded the Tn*4401*a isoform containing a 100-bp deletion (ST 258), and 2 encoded the Tn*4401*c isoform containing a 200-bp deletion upstream of the *bla*_KPC_ gene. These types of transposons tend to evolve by capturing various insertion sequences, as illustrated for the *vanA*-containing Tn*1546* transposon (*40*). For Tn*4401*, three descriptions have been published in which different insertion sequences were present upstream of *bla*_KPC-2_ (*22**–**24*). None of these atypical structures were found in our strains. Observation of Tn*4401* on different plasmids further supports the hypothesis that this transposon contributes to the mobilization and dissemination of the *bla*_KPC_ genes.

Our analysis of several *K. pneumoniae* isolates from 5 geographic origins indicates the spread of different clones that were harboring different plasmids but with an identical genetic structure, Tn*4401*, that sustained a *bla*_KPC_ gene acquisition, which could likely be at the origin of the worldwide spread of this emerging resistance gene. Finally, taken together, our findings and those of recent studies report a major KPC-producing clone with ST 258, even if novel ST types could also be evidenced, especially from Colombia. Our data suggest that KPC genes benefit all molecular ingredients (transposon location, self-transferable plasmids, efficient STs) by facilitating their rapid spread to *K. pneumoniae* and other bacterial species.
